# Hypertension, smoking, and preexistence of multiple cardiac risk factors correlate with carfilzomib-induced cardiovascular adverse events in a racially diverse population

**DOI:** 10.3389/fcvm.2023.1129943

**Published:** 2023-06-08

**Authors:** Stacey Doran, Manu Mysore, Seyed Ebrahim Kassaian, Ethan Kotloff, Farin Kamangar, Ashkan Emadi, Jummai Apata, Brian Barr

**Affiliations:** ^1^ National Cancer Institute, Bethesda, MD, United States; ^2^ Department of Medicine, Division of Cardiovascular Medicine, University of Maryland School of Medicine, Baltimore, MD, United States; ^3^ Medstar Heart and Vascular Institute, Medstar Washington Hospital Center, Washington, DC, United States; ^4^ Department of Biology, School of Computer, Mathematical, and Natural Sciences, Morgan State University, Baltimore, MD, United States; ^5^ University of Maryland Greenebaum Comprehensive Cancer Center, Baltimore, MD, United States; ^6^ Department of Medicine, University of Maryland School of Medicine, Baltimore, MD, United States; ^7^ Department of Pharmacology, University of Maryland School of Medicine, Baltimore, MD, United States; ^8^ Center for Urban Health Disparities Research & Innovation, Morgan State University, Baltimore, MD, United States

**Keywords:** multiple myeloma, carfilzomib, cardiotoxicity, race, cardio-oncology, CVAE

## Abstract

**Background:**

Use of the proteasome inhibitor carfilzomib has become a standard of care in patients with relapsed/refractory multiple myeloma. An association between carfilzomib and cardiovascular adverse events has been well documented, but this had not been investigated in a racially diverse population. Black patients in particular are underrepresented in the reported outcomes of treatment with carfilzomib.

**Objective:**

The purpose of this study was to identify risk factors for carfilzomib-associated cardiovascular events in a diverse, single-center population.

**Methods:**

We conducted a retrospective review of 161 patients with multiple myeloma treated with carfilzomib between 2011 and 2020 at the University of Maryland Medical Center. Over half (86) were Black patients, with the remainder (75) being White patients. We did a multivariate analysis to determine risk factors for developing cardiovascular events during treatment with carfilzomib.

**Results:**

There was no statistically significant association with cardiotoxicity and race, gender, or age at first dose of carfilzomib. In multivariable analysis, patients with history of hypertension had a higher risk of cardiotoxicity [adjusted odds ratio (OR): 2.5; 95% CI: 1.1–5.9; *P* = 0.03] as did those with a history of smoking [OR: 2.8; 95% CI: 1.3–6.4; *P* = 0.01].

**Conclusions:**

Here we report the largest cohort of Black patients treated with carfilzomib as yet reported. The results of this single center retrospective study show history of hypertension and smoking are associated with carfilzomib associated cardiotoxicity in a diverse patient population. There is a need for well-designed prospective studies enrolling a diverse population to investigate potential interventions to prevent carfilzomib-associated cardiotoxicity.

## 1. Introduction

Multiple myeloma (MM) is a plasma cell neoplasm that accounts for approximately 1.8% of all cancers and 10% of hematologic malignancies in the United States (1). Significant disparities exist between racial groups, with Black patients having a more than two-fold increase in incidence and younger age at diagnosis compared to White patients (2). Recent advances in anti-myeloma drug therapies, including the introduction of immunomodulatory drugs (IMiDs), proteasome inhibitors, and CD38-targeting monoclonal antibodies, have led to significantly improved survival outcomes and quality of life for MM patients, though these gains have been unevenly beneficial to White patients (3).

Carfilzomib is a 2nd-generation proteasome inhibitor that received approval by the US Food and Drug Administration in 2012. It is used in combination with dexamethasone with or without an IMiD for treatment of patients with relapsed or refractory MM (RRMM) who have progressed after one or more lines of therapy (4). While phase III clinical trials have demonstrated significant improvements in progression free survival (PFS) and overall survival (OS) with carfilzomib-based regimens, there has been increasing recognition of carfilzomib-associated cardiovascular adverse events (CVAE), particularly heart failure, arrhythmias, and severe hypertension (5–7). In a systematic review and meta-analysis of 24 prospective studies of patients receiving carfilzomib for MM (*n* = 2,594), Waxman et al. reported all-grade and high-grade CVAE rates of 18.1% and 8.2%, respectively, with hypertension (12.2%) and heart failure (4.1%) being the most common types of CVAE (8). Multiple further trials have supported these findings (9, 10). The underlying mechanism of carfilzomib-associated cardiotoxicity remains unknown, but proposed mechanisms include epoxyketone-generated oxidative stress on cardiac myocytes and endothelial dysfunction (11).

While the association between carfilzomib and clinically significant CVAEs has been well established, risk factors that predispose certain patients to developing cardiotoxicity remain unclear. Recent studies have hypothesized that pre-existing cardiac disease (cardiomyopathy, valvular, coronary artery disease), prior and/or concurrent cardiotoxic agents, and age greater than 75 confer a higher risk for carfilzomib-associated cardiotoxicity (12). However, poor accrual of Black patients to MM trials (13) has led to limited data on effect of race on CVAE. We conducted a retrospective study of a diverse population that included a substantial Black population at a tertiary care center to identify factors that confer a higher risk of carfilzomib-associated cardiotoxicity.

## 2. Materials and methods

### 2.1. Patients

A total of 175 patients with MM treated with carfilzomib between November 2011 and July 2020 at the University of Maryland Greenebaum Comprehensive Cancer Center were retrospectively screened. Patients were initially identified by search of the pharmacy dispensing database, and then electronic medical records were reviewed to confirm carfilzomib administration. All patients who had received at least one dose of carfilzomib, either as a single agent or in combination therapy, were included. Of 175 patients identified on review of pharmacy records, three were excluded due to missing records not verifying receipt of carfilzomib, leaving a total of 172 patients. Medical records were then reviewed and baseline characteristics, adverse treatment effects, and echocardiogram data were recorded to evaluate for cardiotoxicity. Race was determined by patient's self-reporting. The study was approved by the University of Maryland Baltimore (UMB) Institutional Review Board (IRB).

### 2.2. Cardiovascular adverse events

Cardiovascular adverse events (CVAEs) were defined as a patient having at least one of the following conditions after treatment with at least one dose of carfilzomib: left ventricular ejection fraction (LVEF) of <53% for patients without a baseline echocardiogram or the combination of LVEF of <53% with a decrease of at least 10% from baseline when baseline LVEF was known [definition of abnormal as per the American Society of Echocardiography (14)], new arrhythmia, new myocardial infarction, new pulmonary hypertension, new symptomatic dyspnea with elevated N-terminal (NT)-pro brain natriuretic peptide, newly diagnosed or worsened hypertension (defined as requiring either new medication or increase in dose of pretreatment medications).

### 2.3. Statistical analysis

Statistical analysis was conducted using Stata 14 software (15). Baseline characteristics of the patients were compared by race using independent *t*-tests for continuous variables and chi-square tests for categorical variables. Likewise, predictors of cardiotoxicity among patients were compared using independent *t*-tests for continuous variables and chi-square tests for categorical variables. Bivariate and multivariable logistic regression models were fitted to identify potential predictors of cardiotoxicity in the patient sample. Odds ratios (OR) and 95% confidence intervals (95% CI) were reported based on bivariate (unadjusted) or multivariable (adjusted) logistic regression analysis.

## 3. Results

A total of 172 patients treated with at least one dose of carfilzomib were identified. The racial breakdown of the identified patients was 86 Black patients, 75 white patients, 6 Asian patients, and 5 other/unknown race patients. Due to the small number of Asian patients and other race patients, the statistical analyses were then run on the 161 Black and White patients.

### 3.1. Baseline characteristics

The baseline patients' characteristics are shown in Table 1. Of 161 eligible patients of our cohort, 59% of Black patients and 32% of White patients were female (*P* = 0.001). The median age at first carfilzomib dose was 59 years. Ninety patients (56%) were >60 years old at time of carfilzomib initiation, with White patients being significantly older (mean age: 62 vs. 58, *P* = 0.009). Prior to treatment, 87% of patients had at least one and 57% had at least 2 cardiovascular risk factors (diabetes, hypertension, hyperlipidemia, smoking, obesity) without significant difference between races. However, White patients had higher frequency of history of at least one diagnosed cardiovascular disease (coronary artery disease, heart failure, valvular disease, pulmonary hypertension and arrhythmia) (26.7 vs. 7.0%, *P* = 0.001) which was mostly attributed to history of coronary artery disease.

**Table 1:** Baseline characteristics of patients by race.

Baseline characteristics	Number of patients *n* (column%)			
	Black *n* = 86 (53.4)	White *n* = 75 (46.6)	Total *n* = 161 (100)	*P*-value^†^
**Demographic Information**				
Female sex	51 (59.3)	24 (32.0)	75 (46.6)	0.001
Mean age at multiple myeloma diagnosis (SD)	54 (10.4)	59 (9.2)	56 (10.1)	0.002
Median age at multiple myeloma diagnosis	55	59	58	
Mean age at 1st dose of carfilzomib (SD)	58 (10.5)	62 (9.1)	60 (10.1)	0.009
Median age at 1st dose of carfilzomib	59	64	61	
**Past medical history**				
Hypertension	54 (62.8)	39 (52.0)	93 (57.8)	0.167
Hyperlipidemia	23 (26.7)	28 (37.3)	51 (31.7)	0.150
Smoking	38 (44.2)	31 (41.3)	69 (42.9)	0.715
Diabetes mellitus	22 (25.6)	12 (16.0)	34 (21.1)	0.137
Coronary artery disease	0 (0.0)	12 (16.0)	12 (7.5)	<0.001
Pulmonary hypertension	1 (1.2)	1 (1.3)	2 (1.2)	0.922
Congestive heart failure	1 (1.2)	2 (2.7)	3 (1.9)	0.481
Cardiac valve disease	0 (0.0)	3 (4.0)	3 (1.9)	0.061
Arrhythmia	5 (5.8)	10 (13.3)	15 (9.3)	0.102
Chronic kidney disease	15 (17.4)	17 (22.7)	32 (19.9)	0.407
**Medical conditions**				
Uncontrolled BP (Systolic ≥140 mmHg or Diastolic ≥90 mmHg at time of 1st dose of carfilzomib	18 (20.9)	21 (28.0)	39 (24.2)	0.296
Mean BMI at time of 1st dose of carfilzomib (SD)	31.4 (8.0)	29.6 (6.4)	30.6 (7.3)	0.123
Median BMI at time of 1st dose of carfilzomib	30.1	28.6	29.0	
Obese (BMI ≥ 30)	43 (51.2)	28 (38.9)	71 (45.5)	0.124
**Baseline echocardiogram findings**				
Baseline echocardiogram obtained	71 (82.6)	69 (92.0)	140 (87.0)	0.076
Mean baseline LV EF (SD)	0.61 (0.07)	0.60 (0.07)	0.61 (0.07)	0.332
Baseline LV EF ≥50%	68 (95.8)	66 (95.6)	134 (95.7)	0.971
Baseline LV EF ≥40%	71 (100.0)	68 (98.5)	139 (99.3)	0.309
LVH	10 (14.7)	11 (16.2)	21 (15.4)	0.812
Diastolic dysfunction	15 (22.7)	9 (14.1)	24 (18.5)	0.203

SD, standard deviation; BMI, body mass index; BP, blood pressure; LV EF, left ventricular ejection fraction; LVH, left ventricular hypertrophy.

^†^ *P* values were based on the independent *t*-test for continuous variables and on the chi-square test or the Fisher exact test for categorical variables without accounting for missing data.

There were 24% of patients with a history of hypertension (prior diagnosis of hypertension in the medical chart and/or on current anti-hypertensive therapy) and 45.5% had obesity (BMI  >  30 kg/m^2^) with no significant differences noted between White patients and Black patients. Baseline echocardiogram assessment showed mean LVEF to be 61%, and although 15.4% of patients had left ventricular hypertrophy and 18.5% had diastolic dysfunction, only 7 (4.3%) patients had LV systolic dysfunction (LVEF < 50%). There were no significant differences in baseline echocardiogram findings between Black and White patients. While most (87%) patients had a baseline echocardiogram, there was a non-significant trend showing that more White patients had a baseline echocardiogram completed when compared to Black patients (92% vs. 82.6%, *P* = 0.07).

### 3.2. Cardiotoxicity

A total of 46 (28.6%) patients met at least one criterion for CVAE as defined in section 2.2; 11 of the patients met more than one criterion. The observed CVAE criteria were decreased LVEF (*n* = 20), new dyspnea with elevated pro-NT-BNP (*n* = 19), new or worsened hypertension (*n* = 11), new arrythmia (*n* = 5), myocardial infarction (*n* = 2), and new pulmonary hypertension (*n* = 1).

There were no statistically significant differences in CVAE occurrence between Black (*n* = 23, 27%) and White (*n* = 23, 31%) patients.

### 3.3. Bivariate and multivariable analysis

In bivariate analysis, there were no significant differences in CVAEs between patient demographics such as race, gender and age at initial multiple myeloma diagnosis or first Carfilzomib dose (Table 2).

**Table 2:** Bivariate analyses of potential predictors of CVAEs.

Baseline characteristics	CVAEs *n* (row %)		
	No *n* = 115 (71.4)	Yes *n* = 46 (28.6)	*P*-value^†^
**Demographic information**			
Female	50 (66.7)	25 (33.3)	0.212
Male	65 (75.6)	21 (24.4)	
Black	63 (73.3)	23 (26.7)	0.583
White	52 (69.3)	23 (30.7)	
Mean age at multiple myeloma diagnosis (SD)	56 (10.8)	58 (7.8)	0.159
Median age at multiple myeloma diagnosis	57	58	
Mean age at 1st dose of carfilzomib (SD)	59 (10.9)	62 (7.5)	0.184
Median age at 1st dose of carfilzomib	61	62	
**Past medical history**			
**Hypertension**			
No	56 (82.3)	12 (17.7)	0.009
Yes	59 (63.4)	34 (36.6)	
**Hyperlipidemia**			
No	78 (70.9)	32 (29.1)	0.830
Yes	37 (72.5)	14 (27.5)	
**Smoking**			
No	73 (79.3)	19 (20.7)	0.010
Yes	42 (60.9)	27 (39.1)	
**Diabetes mellitus**			
No	94 (74.0)	33 (26.0)	0.160
Yes	21 (61.8)	13 (38.2)	
**Coronary artery disease**			
No	109 (73.1)	40 (26.9)	0.088
Yes	6 (50.0)	6 (50.0)	
**Pulmonary hypertension**			
No	114 (71.7)	45 (28.3)	0.500
Yes	1 (50.0)	1 (50.0)	
**Congestive heart failure**			
No	113 (71.5)	45 (28.5)	0.854
Yes	2 (66.7)	1 (33.3)	
**Cardiac valve disease**			
No	114 (72.1)	44 (27.9)	0.140
Yes	1 (33.3)	2 (66.7)	
**Arrhythmia**			
No	106 (72.6)	40 (27.4)	0.304
Yes	9 (60.0)	6 (40.0)	
**Chronic kidney disease**			
No	96 (74.4)	33 (25.6)	0.092
Yes	19 (59.4)	13 (40.6)	
**Risk factors**			
History of 2 or more cardiovascular diseases*			
No	112 (72.3)	43 (27.7)	0.236
Yes	3 (50.0)	3 (50.0)	
**History of 1 or more cardiovascular diseases**			
No	99 (73.3)	36 (26.7)	0.223
Yes	16 (61.5)	10 (38.5)	
**History of 2 or more cardiovascular risk factors** **			
No	55 (79.7)	14 (20.3)	0.044
Yes	60 (65.2)	32 (34.8)	
**History of 1 or more cardiovascular risk factors**			
No	17 (80.9)	4 (19.1)	0.300
Yes	98 (70.0)	42 (30.0)	
**History of 2 or more cardiovascular problems** ^#^			
No	52 (80.0)	13 (20.0)	0.048
Yes	63 (65.6)	33 (34.4)	
**History of 1 or more cardiovascular problems**			
No	16 (84.2)	3 (15.8)	0.189
Yes	99 (69.7)	43 (30.3)	
**Medical conditions**			
**Uncontrolled systolic BP (≥140 mmHg) at time of 1st dose of carfilzomib**			
No	94 (76.4)	29 (23.6)	0.020
Yes	21 (56.8)	16 (43.2)	
**Uncontrolled diastolic BP (≥90 mmHg) at time of 1st dose of carfilzomib**			
No	105 (72.4)	40 (27.6)	0.637
Yes	10 (66.7)	5 (33.3)	
**Uncontrolled BP (Systolic ≥140 mmHg or Diastolic ≥90 mmHg at time of 1st dose of carfilzomib**			
No	92 (75.4)	30 (24.6)	0.048
Yes	23 (59.0)	16 (41.0)	
Mean BMI at time of 1st dose of carfilzomib (SD)	31.1 (7.2)	29.2 (7.7)	0.145
Median BMI at time of 1st dose of carfilzomib	29.8	28.1	
**Obese (BMI ≥30)**			
No	57 (67.1)	28 (32.9)	0.150
Yes	55 (77.5)	16 (22.5)	
**Baseline echocardiogram findings**			
**Baseline echocardiogram obtained**			
No	79 (74.5)	27 (25.5)	0.227
Yes	36 (65.4)	19 (34.6)	
Mean baseline LV EF (SD)	0.61 (0.07)	0.61 (0.07)	0.860
**Baseline LV EF ≥50%**			
No	4 (66.7)	2 (33.3)	0.760
Yes	97 (72.4)	37 (27.6)	
**Baseline LV EF ≥40%**			
No	1 (100.0)	0 (0.0)	0.533
Yes	100 (71.9)	39 (28.1)	
**Baseline LVH**			
No	84 (73.0)	31 (27.0)	0.299
Yes	13 (61.9)	8 (38.1)	
**Baseline diastolic dysfunction**			
No	74 (69.8)	32 (30.2)	0.614
Yes	18 (75.0)	6 (25.0)	

SD, standard deviation; BMI, body mass index; BP, blood pressure; LV EF, left ventricular ejection fraction; LVH, left ventricular hypertrophy.

* Cardiovascular diseases: coronary artery disease, congestive heart failure, cardiac valve disease, arrythmia, pulmonary hypertension.

** Cardiovascular risk factors: diabetes, hypertension, hyperlipidemia, smoking, obesity/BMI 30+.

^#^ Cardiovascular problems: diseases + risk factors.

^†^ *P* values were based on the independent *t*-test for continuous variables and on the chi-square test or the Fisher exact test for categorical variables without accounting for missing data.

**Figure 1:**
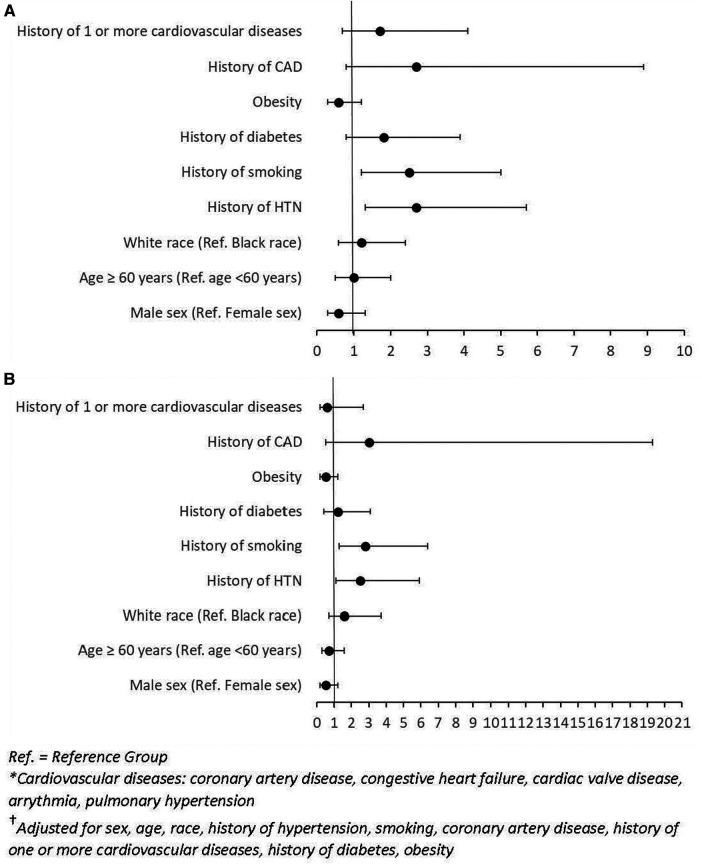
(**A**) Forest plot of odds ratios (OR) and 95% confidence intervals (CI) for unadjusted logistic regression of factors associated with CVAEs. (**B**) Forest plot of odds ratios (OR) and 95% confidence intervals (CI) for adjusted logistic regression of factors^†^ associated with CVAEs.

In multivariable analysis, patients with history of hypertension had a higher risk of CVAEs [adjusted odds ratio (OR): 2.5; 95% CI: 1.1–5.9; *P* = 0.03]. History of smoking was also a significant predictor of cardiotoxicity [OR: 2.8; 95% CI: 1.3–6.4; *P* = 0.01] (Figure 1A). Multivariable analysis of our patient cohort did not show that race, sex, age, or baseline echocardiogram findings were significant predictors of cardiotoxicity when controlling for confounding variables (Figure 1B).

## 4. Discussion

Use of carfilzomib in combination therapy in patients with RRMM has resulted in significant improvement in progression free and overall survival in a difficult to treat patient population. However, this progress in survival has come at the expense of increased cardiovascular adverse events. In 2019, the PROTECT trial (10), a prospective, observational study of risk factors and outcomes in patients with RRMM initiating proteasome-inhibitor therapy, showed that CVAE occurred in 51% of patients treated with carfilzomib and that patients who experienced CVAE had significantly decreased PFS and OS. Observed CVAE included hypertension, arrhythmia, heart failure, ischemic heart disease, cardiomyopathy, thromboembolic events, pulmonary hypertension, and sudden cardiac death. In prior studies, there was noted clinical benefit in measuring either NT-proBNP or BNP as baseline elevations in natriuretic peptides were found to be predictive of CVAE in patients undergoing carfilzomib therapy; however chemotherapy modification in the PROTECT trial was not based on natriuretic peptide levels alone. However, it was deemed that in patients with multiple CV risk factors and elevated natriuretic peptide levels, comprehensive cardiac monitoring and practice based adjustments such as the addition of diuretics or adding to an anti-hypertensive regimen should be performed (10).

As opposed to boronic acid-based reversible proteasome inhibitors such as bortezomib and ixazomib, carfilzomib's structure contains an epoxyketone as the active moiety making it an irreversible proteasome inhibitor through formation of double covalent bonds between the epoxyketone pharmacophore and proteasome (16). Even though the comprehensive mechanism of carfilzomib-associated cardiotoxicity remains unknown, the suggested mechanisms include epoxyketone-generated oxidative stress on cardiac myocytes, endoplasmic reticulum stress, accumulation and cross linking of ubiquinated proteins, and endothelial dysfunction via irreversible covalent bonding. In particular, inhibition of ongoing proteasome-dependent sarcomeric protein turnover appears to be the mechanism of induced apoptosis and cell death (11). Further studies have also shown that combination of doxorubicin with carfilzomib induce more additive cardiotoxicity with further research being performed to address the utility of iron chelator dexrazoxane on toxic concentrations of carfilzomib therapy (17).

In the United States, significant racial disparities exist in patients with MM (2, 3). Compared to White patients, Black patients have more than twice the risk of being diagnosed with MM for unclear and likely multifactorial reasons. While on a population level Black patients have worse outcomes, a recent matched cohort study showed that when Black patients receive equal treatments they may have better 5-year survival outcomes than non-Hispanic White patients (18). Poor accrual of Black patients in clinical trials makes it difficult to delineate causes of disparity on a treatment level. A recent review of accrual of Black patients in multiple myeloma trials found that out of 10,157 patients enrolled in 19 clinical trials, a mere 405 (4%) were Black patients despite the high incidence of disease in the studied population (13). Even within just the population enrolled in the United States, Black patients were 18% of the enrolled population and in 17 of 19 examined studies were <6% of the population. This makes efforts to examine the outcomes of Black patients all the more important to address and correct disparities.

In our retrospective study, we have identified preexisting hypertension, history of smoking, and preexistence of two or more cardiovascular risk factors as increasing risk of developing CVAEs while on carfilzomib. Our single-center, retrospective study of 161 patients is, to our knowledge, a description of the single largest cohort of Black patients to receive carfilzomib, with a diverse group of 86 (53%) Black and 75 (47%) White patients. Prior analysis has shown that Black patients have a higher lifetime risk of hypertension than White adults and specifically, the Multi-Ethnic Study of Atherosclerosis (MESA) has shown that 40 year risk of developing hypertension among adults is higher specifically among Black patients (93%) when compared to White patients (86%). Even more, among adults greater than or equal to 30 years of age, it is noted that higher systolic blood pressure increases the risk for cardiovascular disease including myocardial infarction, heart failure, stroke, and peripheral arterial disease (19). Our study likely was not powered to note a true difference between Black and White patients in terms of risk factors (including hypertension) that predict risk of developing cardiotoxicity for which a larger study would be beneficial. On the other hand, it is notable that risk factors (such as hypertension, prior history of smoking, and/or history of at least two or more cardiovascular risk factors) predict cardiotoxicity regardless of race in our study population.

We included all patients who had received at least one dose of carfilzomib as a part of any treatment regimen. National Comprehensive Cancer Network (NCCN) guidelines recommend carfilzomib to be given as a part of doublet or triplet therapy (20). When given as doublet therapy with dexamethasone, carfilzomib is given once or twice weekly and at higher doses. Triplet therapy is with dexamethasone and a third drug such as lenalidomide, cyclophosphamide, daratumumab, or pomalidomide; dosing in these regimens is weekly and lower than in doublet therapy. Dosing also may be modified at oncologist discretion based on patient tolerance to side effects. One limitation of this study is that we were not able to consider factors such as which regimen the patient was treated with, dosing schedule, or total cumulative dose of carfilzomib, all of which may be factors in development of CVAEs.

This study had other limitations. For unclear reasons Black women were overrepresented compared to Black men. This could mean that any risk factors that are enhanced in Black men may have been attenuated. In terms of imaging, echocardiograms were read by a single reader in a tertiary care center laboratory that were not further verified by a CORE lab. Parameters such as diastolic dysfunction were noted as data points only if noted on an echocardiogram report and not reclassified by independent analysis. Lastly, our study may not be powered to assess various other risk factors for cardiotoxicity.

Early recognition of patients at high risk of CVAE from carfilzomib therapy is critical. Patients at high risk should be considered for a comprehensive cardio-metabolic assessment including possible referral to a cardio-oncologist prior to initiation of therapy. Further studies on whether interventions such as tighter blood pressure control regardless of racial differences can impact outcomes remain to be done. Lastly, it is critical that future studies include more diverse groups of patients so that future advances lead to more equitable improvements in outcomes.

## Data Availability

The raw data supporting the conclusions of this article will be made available by the authors, without undue reservation.
